# Porcelain Gallbladder: Often an Overlooked Entity

**DOI:** 10.1055/s-0037-1606546

**Published:** 2017-09-14

**Authors:** Sohail Iqbal, Sarfraz Ahmad, Usman Saeed, Mohammed Al-dabbagh

**Affiliations:** 1 Department of Cardiac Imaging, North West Heart Centre, Manchester, United Kingdom; 2 Radiology Department, Royal Blackburn Hospital, ELHT, Blackburn, United Kingdom; 3 Radiology Department, Colchester General Hospital, CHUFT, Colchester, United Kingdom

**Keywords:** porcelain gallbladder, cancer, diagnosis, mural calcification

## Abstract

**Background** Porcelain gallbladder (GB) is a rare but potentially premalignant condition with minimal symptoms. Accident and Emergency (A&E) departments often tend to investigate abdominal pain through plain radiographs, which are occasionally reported by radiologists, thereby leaving behind few uncommon conditions, such as porcelain gallbladder unreported.

**Objectives** We present three cases of porcelain GB in which initial diagnosis was not considered due to the presence of various other calcifications in the upper abdomen.

**Methods** In A&E, plain abdominal X-rays were routinely performed in all three patients to investigate nonspecific postprandial abdominal pain. Although GB calcification was easy to diagnose on plain films, it was initially overlooked to be a cause of the symptoms and later was diagnosed on abdominal CT scans, performed for further evaluation.

**Results** Abdominal X-rays revealed thin curvilinear calcification in the GB wall, partially calcified neck and body, and gall stones. CT scan confirmed porcelain GB in all three patients.

**Conclusion** Gallbladder mural calcification is a rare cause of nonspecific abdominal pain, which is often overlooked on plain abdominal X-rays causing missed diagnosis. The association of porcelain GB with adenocarcinoma entails special emphasis on timely diagnosis and prompt management.

With the advancement of imaging modalities, plain film radiology is increasingly overlooked. However, its importance cannot be denied in investigating acute abdominal conditions and diagnosing various causes of calcifications, which can be pathognomonic of certain chronic abdominal diseases. Although plain abdominal radiographs are often diagnostic in depicting majority of renal stones and calcifications of blood vessels and lymph nodes, these rarely demonstrate gallbladder (GB) stones and its mural calcification.

Porcelain GB is a complete or partial calcification of the entire GB wall thickness or its mucosal layer that is visualized on plain abdominal X-ray as a thin curvilinear or speckled calcification in right upper quadrant (RUQ) or more precisely the gallbladder fossa. It is often associated with gallstones, which are usually radiolucent. Ultrasound scan (USS) can demonstrate an echogenic thick shadowing in the GB fossa making it difficult to differentiate from emphysematous cholecystitis. Computed tomography (CT) scan with three-dimensional (3-D) reconstruction is considered highly efficient in diagnosing this condition.

## Case Reports

We present three cases of porcelain GB with abdominal pain. The first case was a middle-aged Mediterranean male patient who presented with intermittent postprandial pain on several occasions in A&E. His plain abdominal film revealed a faint thin curvilinear calcification in RUQ. Subsequently, he had an abdominal CT scan that confirmed the diagnosis of porcelain GB by demonstrating a heavily calcified neck and body of the GB.

The remaining two patients were English females in their sixties. One patient was being treated for renal colic and was incidentally diagnosed to have a porcelain GB in addition to left renal stone and abdominal aortic calcification on plain abdominal X-rays that was later confirmed on CT scan. The other patient was investigated for RUQ abdominal pain and had plain abdominal X-rays that showed porcelain GB with cholelithiasis. Later on, CT scan confirmed the diagnosis.

### Imaging Findings

**Fig. 1:**
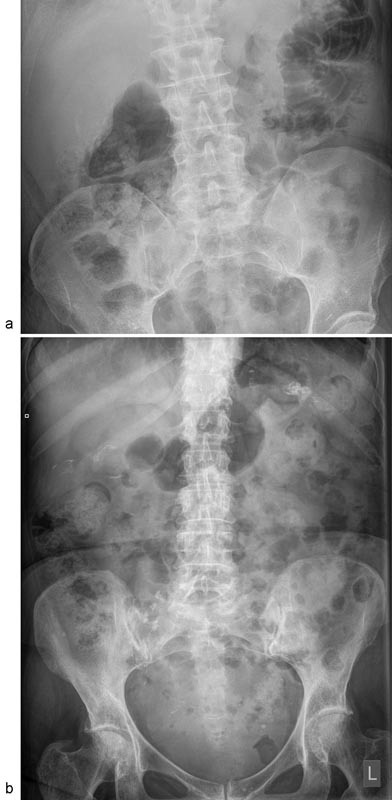
( **A** ) Plain film showing thin curvilinear calcification in the GB wall. ( **B** ) Heavily but partially calcified body and neck of the GB. GB, gall bladder.

**Fig. 2:**
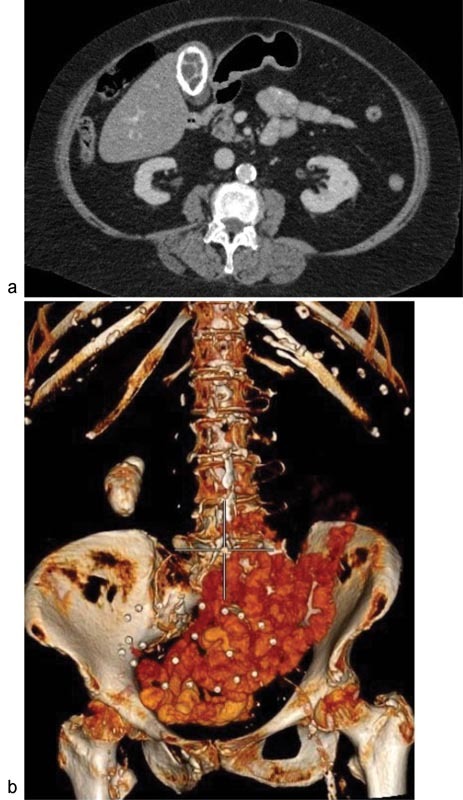
( **A** ) CT scan of the first patient confirming porcelain GB. ( **B** ) A 3-D reconstruction of the CT scan. CT, computed tomography.

**Fig. 3:**
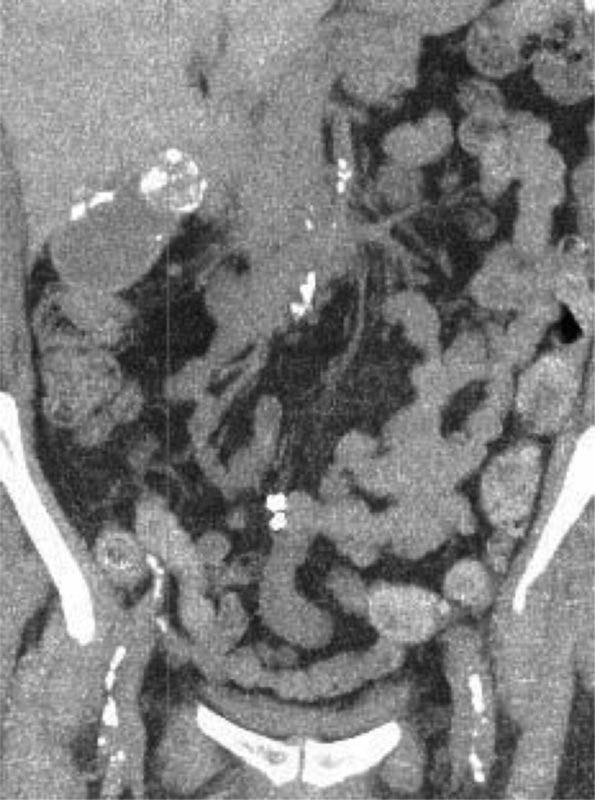
Calcified GB along with stone in the neck of the GB. GB, gall bladder.

Porcelain GB is easy to recognize because of its characteristic plain film thin curvilinear GB wall calcification in the GB fossa ( Fig. 1A ) or heavily but partially calcified body and neck of the GB ( Fig. 1B ). CT scan is far superior in recognizing this condition ( Fig. 2A ) with 3-D reconstruction ( Fig. 2B ). It is often associated with stone in the neck of the gall bladder ( Fig. 3 ).

## Discussion

Porcelain GB is a calcified GB having characteristic bluish appearance and brittle texture on macroscopic examination. Histopathologically, it is divided into selective mucosal and complete intramural subtypes based on the distribution of dystrophic calcification across the GB wall. Autopsy specimens have shown an incidence of up to 0.8%, with female to male preponderance of 5:1. 1

It is thought to result from chronic cholecystitis and is associated with cholelithiasis in 95% of cases. Female gender, cholesterol cycling, hormonal factors, bacterial infections, and ethnicity are considered common risk factors for the formation of gall bladder stones. 2 Porcelain GB may be associated with epithelial hyperplasia, epithelial dysplasia, and intestinal and gastric metaplasia, 3 which can lead to malignancy. A variable risk of malignancy has been reported. 4 Recent studies have shown 6% increased risk of developing adenocarcinoma in selective mucosal calcification type as compared with complete intramural type. 5 6

Majority of patients are asymptomatic; however, few may present with mild symptoms of biliary disease such as indigestion and postprandial pain. The thickening and calcification of GB ultimately render it nonfunctional, which can be seen on oral cholecystogram and technetium-99m hepato imido diacetic acid (HIDA) radionuclide uptake imaging. On plain radiograph or CT scan, a typical GB fossa calcification can be visualized in patients with radio opaque gall stones and porcelain GB demonstrating curvilinear calcifications of a segment or the entire wall. However, CT is more sensitive than conventional radiographs. Although an ultrasound scan (USS) can depict highly echogenic acoustic shadowing with curvilinear structure in the GB fossa, it remains difficult to differentiate porcelain GB from emphysematous cholecystitis, which is more common in diabetic patients and has a reversed male to female ratio of 5:1. 7 CT scan with 3-D reformatting is much superior and sensitive modality to delineate porcelain GB.

Laparoscopic cholecystectomy is preferred over open cholecystectomy in patients with noncomplicated porcelain GB. Recently, single port laparoscopic cholecystectomy has been described through a 2-cm umbilical incision with single incision laparoscopic system-SILS (Covidien; Mansfield, OH) having three 5-mm holes. 8 In complicated patients, open cholecystectomy is the treatment of choice to avoid theoretical risk of tumor seeding.

Porcelain GB is a rare but potentially premalignant condition with minimal symptoms. Therefore, its diagnosis and treatment are still challenging for treating physicians because of high morbidity and mortality associated with the adenocarcinoma of the GB. Radiologists can offer more help through an early detection of the condition.
